# Hunting, Exotic Carnivores, and Habitat Loss: Anthropogenic Effects on a Native Carnivore Community, Madagascar

**DOI:** 10.1371/journal.pone.0136456

**Published:** 2015-09-16

**Authors:** Zach J. Farris, Christopher D. Golden, Sarah Karpanty, Asia Murphy, Dean Stauffer, Felix Ratelolahy, Vonjy Andrianjakarivelo, Christopher M. Holmes, Marcella J. Kelly

**Affiliations:** 1 Virginia Tech, Department of Fish and Wildlife Conservation, Blacksburg, VA, United States of America; 2 Department of Environmental Health, Harvard School of Public Health, Boston, MA, United States of America; 3 Wildlife Health and Health Policy Program, HEAL (Health & Ecosystems: Analysis of Linkages) Wildlife Conservation Society, NY, United States of America; 4 Wildlife Conservation Society Madagascar Program, Antananarivo, Madagascar; 5 Wildlife Conservation Society Asia Program, Vientiane, Laos; University of Queensland, AUSTRALIA

## Abstract

The wide-ranging, cumulative, negative effects of anthropogenic disturbance, including habitat degradation, exotic species, and hunting, on native wildlife has been well documented across a range of habitats worldwide with carnivores potentially being the most vulnerable due to their more extinction prone characteristics. Investigating the effects of anthropogenic pressures on sympatric carnivores is needed to improve our ability to develop targeted, effective management plans for carnivore conservation worldwide. Utilizing photographic, line-transect, and habitat sampling, as well as landscape analyses and village-based bushmeat hunting surveys, we provide the first investigation of how multiple forms of habitat degradation (fragmentation, exotic carnivores, human encroachment, and hunting) affect carnivore occupancy across Madagascar’s largest protected area: the Masoala-Makira landscape. We found that as degradation increased, native carnivore occupancy and encounter rates decreased while exotic carnivore occupancy and encounter rates increased. Feral cats (*Felis species*) and domestic dogs (*Canis familiaris*) had higher occupancy than half of the native carnivore species across Madagascar’s largest protected landscape. Bird and small mammal encounter rates were negatively associated with exotic carnivore occupancy, but positively associated with the occupancy of four native carnivore species. Spotted fanaloka (*Fossa fossana*) occupancy was constrained by the presence of exotic feral cats and exotic small Indian civet (*Viverricula indica*). Hunting was intense across the four study sites where hunting was studied, with the highest rates for the small Indian civet ($\bar{\text{x}}\text{ = }90$ individuals consumed/year), the ring-tailed vontsira (*Galidia elegans*) ($\bar{\text{x}}\text{ = }58$ consumed/year), and the fosa (*Cryptoprocta ferox*) ($\bar{\text{x}}\text{ = }31$ consumed/year). Our modeling results suggest hunters target intact forest where carnivore occupancy, abundance, and species richness, are highest. These various anthropogenic pressures and their effects on carnivore populations, especially increases in exotic carnivores and hunting, have wide-ranging, global implications and demand effective management plans to target the influx of exotic carnivores and unsustainable hunting that is affecting carnivore populations across Madagascar and worldwide.

## Introduction

Carnivores are at great risk from anthropogenic disturbance and habitat degradation due to their extinction-prone characteristics such as large body size, wide-ranging behavior, low density, low recruitment, and specialized diet [1–7]. Carnivore populations worldwide are known to be negatively affected by a number of anthropogenic pressures, including fragmentation, degradation, exotic species, and hunting. Forest loss and fragmentation reduce available habitat and resources, impede gene flow, alter ranging patterns, and lead to increases in exotic species [8–13]. The presence of exotic species has been shown to negatively affect native carnivore behavior, health, and population demographics in multiple habitats worldwide [14–19]. Hunting presents a serious threat to carnivore populations, as has been shown for numerous species across the globe [20–25]. While these studies provide important insights into the effects of anthropogenic pressures on carnivore populations, we need a better understanding of how these pressures act both individually and synergistically on carnivores over a gradient of disturbance across a large spatial area.

An understanding of these pressures and their impacts on carnivore communities is important for developing targeted, effective management strategies for carnivore populations worldwide. These targeted management plans are greatly needed as more than half of the world’s carnivores are currently listed as threatened and an estimated 77% of carnivore species are undergoing population declines [7,26,27]. As forest loss and fragmentation of carnivore habitat increase worldwide, resulting in greater human-carnivore conflict, the rate of carnivore population decline is likely escalate. The conservation of carnivore communities must be a major focus for various conservation organizations and government bodies given that the removal of carnivores from ecosystems can result in trophic cascades, mesopredator release, and the loss of additional ecosystem services and co-occurring species [7]. Carnivora represents a well-studied order of wildlife [28]; however, investigations of sympatric carnivore communities, including the effects of anthropogenic pressures on an entire community, are rare. These community-level investigations are needed, particularly for those poorly represented carnivore families, such as the Eupleridae carnivores of Madagascar [28].

Madagascar has received much conservation attention over the last decade as a result of its high levels of biodiversity and endemism, as well as the increasing anthropogenic pressures threatening it [1,29–32]. On-going trends in forest loss, degradation, and fragmentation pose serious threats to the native wildlife of Madagascar [30,33–35]. In addition, recent research in Madagascar has highlighted the growing threats to wildlife resulting from an influx of exotic species [13,18,36–40] and from unsustainable hunting rates [23,25,41–43]. These various anthropogenic pressures have been shown to negatively affect a number of species of carnivores [13,18,23,25,44,45] and lemurs [23,35,38,39,46,47]. However, our knowledge of how Madagascar’s native wildlife is responding to increases in specific types of anthropogenic pressures remains limited, especially for Madagascar’s little-known carnivores.

Using Madagascar’s carnivore community as a case study, we provide the first investigation of how multiple forms of anthropogenic pressure, which cumulatively result in habitat degradation but individually include fragmentation, exotic species, human encroachment, and hunting, affect carnivore populations. We focused on native carnivores (fosa *Cryptoprocta ferox*, spotted fanaloka *Fossa fossana*, falanouc *Eupleres goudotii*, ring-tailed vontsira *Galidia elegans*, broad-striped vontsira *Galidictis fasciata*, and brown-tailed vontsira *Salanoia concolor*) and exotic carnivores (domestic dog *Canis familiaris*, feral cat *Felis species*, and small Indian civet *Viverricula indica*). Our specific objectives were to: 1) photographically sample wildlife across seven sites with varying levels of degradation, and thus varying levels of fragmentation, exotic species, human presence, and hunting; 2) estimate single-season occupancy and detection for six native and three exotic carnivore species at the individual sites, and across the landscape; 3) identify the covariates (station-level habitat and landscape features, co-occurring native and exotic carnivore species, prey species, and human presence) that have the greatest influence on native and exotic carnivore occupancy and detection; and 4) investigate the effects of hunting pressures (total consumption, trapping, purchasing, and hunting with dogs) on carnivore occupancy and detection at four study sites in the landscape.

## Methods

### Study sites: selection and ranking

**Fig 1:**
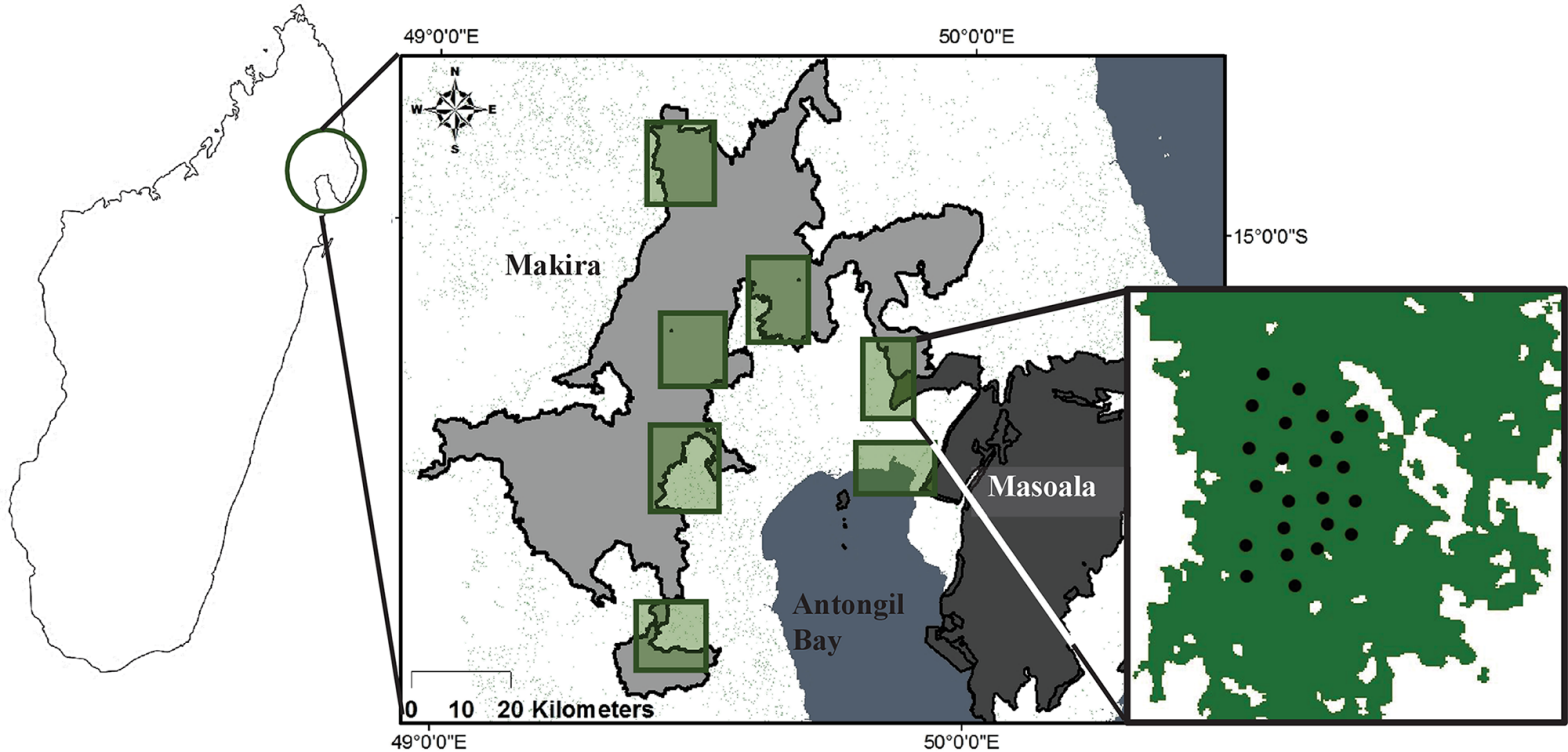
Map of the Masoala-Makira landscape. Our study site map includes the outline of the Masoala (right in light gray) and Makira (left in dark gray) protected areas in which the seven surveys, indicated by green boxes, were conducted including an inset map of one of our camera grids showing the placement of cameras (black dots) across available forest cover (green). Bushmeat surveys across the region occurred from 2005–2011 while photographic surveys occurred from 2008–2012. Names of the study sites and/or villages are withheld as required by IRB due to our bushmeat survey data. Map created by ZJ Farris and Wildlife Conservation Society Madagascar Program staff.

The newly established Makira Natural Park (hereafter Makira) is Madagascar’s largest protected area with 372,470 ha of protected area and 351,037 ha of community management zone [48]. Madagascar’s second largest protected area, Masoala National Park (hereafter Masoala), contains 240,000 ha in protected forest and borders Makira NP to the south-west [31]. This Masoala-Makira protected landscape (Fig 1) is the largest area of contiguous forest and is estimated to have the highest levels of biodiversity in Madagascar, including at least 22 species of lemurs and six species of native carnivores [44,48,49]. Farris et al. [19] provide a review of the native and exotic carnivore community across the Masoala-Makira landscape, including details on body size, weight, IUCN status, diet, and habitat preferences. Despite its expansive size and high level of biodiversity, the Masoala-Makira landscape faces numerous anthropogenic pressures common to Madagascar’s forests, such as hunting [23,25], habitat degradation and fragmentation [48,50,51], and an influx of exotic species [18,39,44].

Across the Masoala-Makira landscape we selected seven study sites with varying levels of degradation within which to establish photographic sampling stations (Fig 1; Table 1). We selected the sites based on the following a-priori criteria acquired from WCS field researchers, local Malagasy people, and/or satellite imagery: 1) presence of logging or clear cutting; 2) presence of local mining; 3) nearest distance to and total amount of forest edge; 4) level of fragmentation; 5) total amount of contiguous forest; 6) accessibility of the site; and 7) presence of human and/or game trails. We sampled sites within the Makira protected area (*n* = 4), within a fragmented reserve near Masoala (*n* = 1), outside the protected area near Makira (*n* = 1) and near the Masoala-Makira corridor (*n* = 1; Fig 1). We quantified levels of degradation and, using a maximum likelihood estimated principle components analysis (PCA), we ranked our seven study sites from least to most degraded based on the Eigen vectors and resulting bi-plots generated from a host of features associated with the following metrics: fragmentation, exotic carnivores, human presence, and hunting. To reduce multicollinearity, we examined correlations among individual variables within our metrics of degradation, and variables that were highly correlated (ǀrǀ > 0.7) were eliminated, thereby reducing the number of covariates and decreasing redundancy in both our PCA and single-season occupancy models. Due to the sensitivity of our hunting data and in compliance with our human subjects protocol, we labelled sites based on their level of degradation (01 = least degraded to 07 = most degraded), rather than using the village or forest names.

**Table 1:** Station-level habitat (50 m radius of camera stations) and landscape (500 m radius buffer surrounding all stations) features (±SE) for the seven study sites, ranked from least degraded (S01) to most degraded (S07), across the Masoala-Makira landscape. Sampling of habitat and landscape features took place from 2008 to 2012.

			Least		Level of Degradation		Most	
Level	Variables/Covariates	Site S01	Site S02	Site S03	Site S04	Site S05	Site S06	Site S07
Station-level habitat	TreeDen (stems ≥5cm / ha) ^a^	1,200 (300)	3,500 (900)	4,100 (1,600)	4,600 (1,700)	4,400 (1,100)	-	3,000 (700)
	BA (stems ≥5cm, m^2^/ha) ^b^	82.00 (10.22)	57.4 (6.11)	22.85 (4.59)	73.54 (13.03)	76.54 (8.48)	-	49.85 (6.35)
	Can Ht (m) ^c^	16.97 (1.95)	12.50 (0.96)	7.48 (0.67)	10.55 (1.23)	12.89 (1.08)	-	9.75 (1.27)
	% Can Cover ^d^	64.15 (5.58)	57.05 (4.89)	62.75 (3.17)	43.52 (6.82)	60.84 (4.09)	-	42.45 (5.14)
	% Understory Cover (0–2 m)	0.50 (0.05)	0.44 (0.04)	0.53 (0.03)	0.46 (0.04)	0.44 (0.05)	-	0.52 (0.04)
Landscape	# Patches ^e^	3	10	22	21	31	116	190
	Largest Patch Index ^f^	60.38	52.33	44.88	51.30	39.90	43.72	50.36
	LSI ^g^	1.04	1.34	2.12	1.95	2.02	3.11	6.76
	%Rainforest	99.94	98.89	94.48	95.19	96.87	96.06	81.07
	%Matrix ^h^	0.05	0.66	4.38	0.59	0.76	0.19	4.07
	Tot Core Rainforest (ha) ^i^	0.88	0.99	0.85	0.87	1.14	0.72	0.59
	Tot Edge (m per ha)	0.03	0.59	1.85	1.53	2.13	3.51	7.89
	Avg. Dist. to Village (km)	10.96	2.80	3.33	2.08	4.82	2.71	1.45
	Avg. Dist. to Edge (km)	1.14	0.68	0.29	0.36	0.34	0.60	0.18

^a^ TreeDen = tree density averaged across all camera stations (*n* = 18–25) for each study site

^b^ BA = average basal area

^c^ Can Ht = average canopy height

^d^ % Can Cover = average percent canopy cover

^e^ #Patches: total number of rainforest, degraded forest, and matrix patches within the camera grid buffer

^f^ Largest patch index: the percentage of total landscape area comprised by the largest rainforest patch

^g^ LSI: landscape shape index or the standardized measure of total edge adjusted for the size of the landscape

^h^ %Matrix: percent matrix defined as non-forest land cover consisting of cultivation, open field, or early succession

^i^ Tot Core Area: total core area defined as the sum of the core areas within the camera grid buffer (accounting for 500m edge depth) of each rainforest patch

### Photographic Sampling

From 2008 to 2012 we established camera grids, consisting of 18–25 camera stations per grid, at each of the seven study sites across the Masoala-Makira landscape (Fig 1). To account for seasonal variation we conducted surveys evenly across each of the three seasonal periods: hot-wet (n = 2 surveys), hot-dry (n = 2 surveys), and cool-wet (n = 3 surveys) [19]. Further, while some of Madagascar’s carnivores have been shown to alter their temporal activity patterns across seasons [19,52], in the case of our surveys across the Masoala-Makira region, seasonal variation was not important for explaining the occupancy or detection of native or exotic carnivores (Farris, unpublished data). We surveyed each of the seven study sites an average of 67 days (Table 2) to provide adequate capture rates for estimating detection probabilities while satisfying population closure assumptions and ensuring model convergence for these carnivore species [13,53], including the more rare and elusive species (ex. brown-tail vontsira). Within each of the seven sites, we spaced camera stations consisting of two digital (Reconyx PC85 & HC500, Wisconsin, USA; Moultrie D50 & D55, Alabama, USA; Cuddeback IR, Wisconsin, USA) and/or film-loaded (DeerCam DC300) remote sensing cameras approximately 500 m apart. This distance was used as it was equal to or greater than the estimated home range of Madagascar’s native carnivores, excluding the fosa [39], thus ensuring independence in photo trap locations and allowing us to measure the true occupancy of these target species. We placed cameras on opposing sides of existing human (0.5–2.0 m wide) and game (< 0.5 m wide) trails, avoiding whenever possible, the establishment of newly cut trails. Cameras were slightly offset to prevent mutual flash interference and were paired with an opposing brand or model of camera to compensate for inefficiency in detection speed, flash, or photo quality of various camera models. We checked cameras every 5–10 days to change batteries, memory cards and/or film, and to ensure proper functioning. We placed cameras 20–30 cm off the ground, allowed them to run for 24 h, and used no bait or lure.

**Table 2:** Summary of survey effort, lemur species richness and abundance, and encounter rates for six native and three exotic (bold) carnivores, birds, and small mammals at each of the seven study sites.

		Least		Level of Degradation		Most	
Study site	Site S01	Site S02	Site S03	Site S04	Site S05	Site S06	Site S07
Survey Dates	Mar 2009–May 2009	Sept 2008–Nov 2008	Aug 2009–Oct 2009	Jun 2011–Aug 2011	Mar 2011–May 2011	Nov 2009–Jan 2010	Dec 2010–Feb 2011
# of Camera Stations	20	25	19	23	23	18	24
Trap Nights	1050	1257	1067	1462	1509	881	1570
Elevation (m)	1000–1400	350–690	380–550	21–385	324–786	580–820	93–507
Fosa TS*	0.41 (0.41)	3.01 (0.98)	1.19 (0.30)	1.03 (0.35)	7.15 (1.05)	0.57 (0.20)	1.96 (0.73)
Spotted fanaloka TS	1.03 (0.49)	13.91 (2.64)	0 (0)	0 (0)	5.08 (1.35)	0.18 (0.16)	2.04 (0.36)
Falanouc TS	0 (0)	3.08 (0.89)	0 (0)	2.64 (0.82)	0.33 (0.21)	0.79 (0.27)	0.48 (0.20)
Ring-tailed vontsira TS	0.39 (0.18)	1.33 (0.45)	0.09 (0.09)	1.11 (0.29)	3.75 (1.63)	0.51 (0.37)	0.45 (0.20)
Broad-striped vontsira TS	0.18 (0.13)	2.57 (0.86)	0.19 (0.13)	0.13 (0.13)	0.20 (0.11)	1.31 (0.40)	1.08 (0.37)
Brown-tailed vontsira TS	0 (0)	0.98 (0.30)	0 (0)	0 (0)	0 (0)	0 (0)	0.30 (0.17)
**Domestic dog TS**	0.14 (0.19)	1.97 (1.15)	4.78 (1.77)	14.91 (7.41)	26.06 (4.46)	0.09 (0.08)	19.56 (7.33)
**Feral cat TS**	0.39 (0.19)	0 (0)	0.74 (0.26)	0 (0)	1.32 (0.48)	3.13 (1.18)	0 (0)
Small **Indian civet TS**	0 (0)	0.14 (0.14)	0.10 (0.10)	1.96 (0.74)	0.13 (0.13)	0.12 (0.10)	0.40 (0.16)
Total Bird TS	13.64 (2.76)	62.85 (9.26)	9.22 (1.78)	24.07 (3.93)	23.35 (5.04)	22.61 (3.58)	31.18 (5.48)
Total Small Mammal TS	40.05 (5.30)	42.31 (6.84)	15.15 (3.52)	4.34 (1.20)	4.34 (1.24)	31.59 (4.31)	6.86 (1.50)
Lemur species richness	9	7	3	3	6	NA	4
Total Lemur Abundance**	1.52 (0.11)	0.89 (0.10)	0.45 (0.04)	0.98 (0.17)	0.93 (0.05)	NA	0.45 (0.03)

* TS: trap success is the number of independent photographic capture events of a target species divided by the number of trap nights multiplied by 100

** Relative abundance = number of lemur species (diurnal and nocturnal) observed per km

### Sampling Metrics of Degradation and/or Anthropogenic Pressure

#### Station-level Habitat and Landscape Sampling

To measure station-level habitat features (Table 1) for use in single-season occupancy models, we sampled vegetation at each camera station by walking a 50 m transect in three directions (0, 120, and 240 degrees) starting at each individual camera station. This distance provided a measure of habitat features with an approximately 100 m diameter surrounding each camera station which we believe is sufficient for investigating habitat use for these small-bodied, small home range (< 500 m) carnivores. We estimated canopy height and percent cover at 10 m intervals along each transect. At 25 m and 50 m on each transect we used the point-quarter method [54] to estimate tree density and basal area, recording diameter at breast height (DBH) for any stem/tree with ≥ 5 cm diameter. At 20 m and 40 m we established a 20 m transect running perpendicular to the established 50 m station-level habitat transect and we measured understory cover at three levels (0–0.5 m, 0.5–1.0 m, and 1.0–2.0 m) by holding a 2 m pole perpendicular to the ground at one meter intervals and recording presence (1 = vegetation touching pole) or absence (0 = no vegetation touching pole) of understory cover [55]. We used this sampling array, including the sampling scale, to provide station-level habitat sampling covariates for Madagascar’s carnivores for use in our landscape and site-specific single-season occupancy models. Finally, we also measured trail width (m) and trail type (human, game, or new) as model covariates.

To understand how landscape features varied by site and how they influence carnivore populations, we used Landsat satellite imagery (2004, 2006, and 2009) and classified the following cover types using Erdas Imagine (Intergraph Corporation): rainforest (intact forest with little to no logging present), degraded forest (forest exhibiting disturbance from forest loss, logging, and fragmentation), and matrix (non-forest area exhibiting early succession, cultivation, or open fields for cattle). To create a camera grid buffer that encompassed all camera stations within each of the seven camera grids/sites, we placed a 500 m (landscape level) buffer around individual camera stations, we dissolved the internal individual buffers, and clipped the classified imagery for each of the resulting seven camera grid buffers (each providing an approximately 10–15 km^2^ area) for analysis in FragStats [University of Massachusetts, USA] [56]. For fosa we placed a 2000 m buffer around individual camera stations to extract more meaningful landscape covariates given the estimated home range of this apex carnivore [57].

Using FragStats and the clipped imagery from each camera grid buffer (~10–15 km^2^), we created the following landscape level covariates (Table 1) for use in our single-season occupancy models: 1) number of patches: total number of rainforest, degraded forest, and matrix patches (based on habitat classifications from satellite imagery) within the buffer; 2) largest patch index: the percentage of total buffered area comprised by the largest rainforest patch; 3) LSI (landscape shape index): the standardized measure of total edge adjusted for the size of the buffered area [56]; 4) percent rainforest within the buffered area; 5) percent matrix or non-forest, cultivated area within the buffered area; 6) total rainforest core area: the sum of the core areas (accounting for 500m edge depth) of each rainforest patch within the buffer; and 7) total edge (in meters per hectare) [56]. Further, we estimated an average distance of each camera station to the nearest forest edge (Avg. Dist. to Edge) and to the nearest village (Avg. Dist to Village; Table 1) using satellite imagery and we recorded the elevation at each camera station to use as a covariate.

#### Co-Occurring Species Activity

We defined a single ‘capture event’ as all photographs of a given species within a 30 min time period [58]. To provide a measure of encounter rate for native and exotic carnivores, zebu, bush pigs, small mammals (native and non-native rodents and tenrecs), birds, and humans for comparison across study sites (camera grids) and for use in our single-season occupancy models, we calculated the trap success (TS) for each species by dividing the number of capture events by the number of trap nights at each camera station, minus malfunctions, and multiplied by 100. We defined a trap night as a 24 h period in which at least one of the two cameras at a camera station is functioning properly and is meant to represent animal activity at the station.

To investigate the relationship of lemur prey to carnivore occupancy and to compare lemur species richness and abundance across sites we established three, two kilometer long transects within each camera grid and surveyed each transect five to six times diurnally during the hours of 07:00 to 11:00 and five to six times nocturnally during the hours of 18:00 to 23:00. For each lemur observation we recorded the species, compass direction, distance to observed lemur, height from the ground, number of individuals within the group, and behavior of the individual or group. We were unable to model lemur density in program DISTANCE due to the scarcity of sightings per grid, thus we calculated an index of lemur abundance (# / km) for each camera grid by dividing the number of lemur observations, where lemur groups were counted as a single observation, by the total number of kilometers surveyed both diurnally and nocturnally.

#### Hunting rates

From 2005 to 2011, CDG and his team surveyed 417 households in 26 villages across Makira to estimate annual household consumption rates of bushmeat [23,25,59]. Survey teams made repeated visits to villages and used randomly clustered sampling to select and survey households over the six year period (*mean* = 1 visit/year for 3 years). CDG and his team recruited households over a period of several years and re-surveyed the same households over this time period (see [25] and [59] for details on sampling protocol). For our analyses, we combined two data sets from data being collected simultaneously on different projects (photo capture surveys and household bushmeat surveys) where the two projects overlapped. We only used household bushmeat surveys from villages that bordered our study sites (≤ 5 km from edge of the study site). If any of the seven survey sites did not have hunting surveys from nearby villages we did not use those surveys in our analyses. This provided us with hunting data for seven carnivore species (fosa, spotted fanaloka, falanouc, ring-tailed vontsira, broad-striped vontsira, feral cat, and small Indian civet) from four of our seven study sites (S01, S02, S03, S06). We used the bushmeat data set to calculate the total number of individual animals consumed per village per year for each of the seven carnivore species. We surveyed only a subset of households in each village (S01 = 130; S02 = 239; S03 = 103; S06 = 42). As a result, these estimates of consumption are considered conservative given that they represent the households surveyed and not the total number of households for each village (n = 12–177 households per village). In addition, we included the following sub-categories that contributed to the total consumption: 1) the number of individuals trapped per year; 2) the number of individuals purchased per year; and 3) the number of individuals hunted with domestic dogs. It is very likely that all purchased animals, a very small proportion of total consumption, are sold in the community in which they are harvested [25]. Therefore, it is appropriate to include purchased species as a variable that could affect the local stock of a given species and variation in the number of purchased species indicates a village-level preference for a particular species (a potential proxy for intensity of pressure or targeting). Further, while total consumption may be consistent for a given carnivore species across multiple sites, the way in which the carnivore was obtained (i.e. trapping or hunting with dogs) may vary across these sites and thus provide a different relationship with carnivore occupancy and/or detection across sites. As a result, we used total consumption, as well as the other three sub-categories of total consumption for our analyses. We examined correlations among all hunting covariates (total consumption, number trapped, number purchased, and number hunted with dogs) and removed correlated variables (ǀrǀ > 0.7) from our single-season occupancy analyses (denoted by footnote in results) to eliminate redundancy. Where surveys existed for two villages bordering our study sites we averaged the hunting rates for the two villages to provide a single hunting rate for the study site.

### Carnivore Occupancy Estimation

#### Single-season Occupancy: Landscape and Site Specific

To investigate the effects of each degradation metric on carnivore populations, we conducted three separate single-season occupancy analyses: landscape occupancy, site specific occupancy, and hunting occupancy. To estimate carnivore single-season occupancy and assess the effect of degradation (i.e., fragmentation, exotic carnivores, and human presence) across the landscape (landscape occupancy) and within each of the seven camera grids (site specific occupancy) we created capture histories for each of the six native and three exotic carnivore species using daily capture events to determine the presence (1) or absence (0) of each species at each camera station. We collapsed these daily capture histories into six day occasions to improve model convergence. As a result of the reduced data set for hunting occupancy, we analyzed those sites separately (see below). We analyzed capture histories in program PRESENCE [Patuxent Wildlife Research Centre, USGS, Maryland, USA] [60] to provide an estimate of species occurrence and detection while accounting for spatial variation and variation in detection probability [61]. We used station-level habitat and landscape features, co-occurring species encounter rates (i.e. trap success), prey species encounter rates, and human encounter rates as covariates in our landscape, and site-specific, single-season occupancy models to determine factors that influence native and exotic carnivore occupancy and detection at both landscape and site specific levels. We normalized all covariates before analysis within program PRESENCE.

We used Akaike Information Criterion (AIC) and model selection to rank models [62]. For each carnivore species we reported all competing models (ΔAIC < 2.0), used model averaging to provide an estimate of single-season occupancy and detection, and evaluated the importance and/or effect of covariates on carnivore occupancy and detection using beta estimates from our highest-ranking model. We assessed goodness of fit (GOF) for the most heavily parameterized model for each species’ model set (for both landscape and site specific occupancy) using Pearson’s GOF test (P = 0.05) and evaluated over-dispersion using the ĉ value. For any ĉ value > 3.0 or GOF value > 0.05 (indicating the model did not fit the data well) we used the naïve occupancy estimate (i.e. number of sites capturing the target species out of the total sampling area without accounting for imperfect detection) rather than the estimated value from the model.

#### Occupancy: Hunting

Hunting data were available for only four of our seven study sites. As a result, we conducted an additional single-season occupancy analyses using only hunting rates (hereafter referred to as hunting occupancy) as covariates for these four sites to evaluate how hunting rates (total individuals consumed, trapped, purchased, or hunted with dogs per year) influenced carnivore occupancy and detection across these four study sites. For each carnivore species estimated with our hunting occupancy models we reported all competing models (ΔAIC < 2.0) and evaluated the importance and/or effect of hunting covariates on carnivore occupancy and detection using beta estimates from our highest-ranking model.

#### Ethics Statement

The photographic and line-transect surveys were conducted, with permission, on both private land and within the protected areas of Makira Natural Park and Farankarina reserve and was approved by the Madagascar Ministry of the Environment and Forests (permit No 128/11 and 128/12), the Wildlife Conservation Society Madagascar Program, and Antongil Conservation. These photographic and line-transect surveys were non-invasive and, as a result, no protected species were sampled and no institutional animal care and use (e.g. IACUC) approval was required. The annual bushmeat consumption surveys were approved by the University of California, Berkeley’s Committee on the Protection of Human Subjects (CPHS#2007–2–3) and the Ministry of Water and Forests in Madagascar (#135/09/MEFT/SG/DGEF/DSAP/SLRSE). We also obtained approval from the listed boards to receive oral informed consent from all study participants because there are illiterate members of the population for whom reading consent documents and signing their name would not be possible. As a result, all participants in the bushmeat surveys provided oral consent to participate.

## Results

### Trends in habitat and landscape features

We ranked our seven study sites based on their level of degradation and present the station-level habitat and landscape variables for each of the seven sites (Table 1). Our photographic surveys accumulated a total of 8,854 trap nights (mean = 1,264 per grid) across our seven study sites and captured all six native and three exotic species of carnivores known to occupy the Masoala-Makira landscape [36,44] (Table 2). We found no discernable trend between native carnivore trap success and degradation; however, exotic carnivores showed higher trap success in degraded sites (S05 to S07) compared to less degraded sites (S01 and S02; Table 2). In addition, we found an increase in bird trap success in the little degraded S02 site and small mammal trap success was considerably higher in non-degraded forest compared to degraded sites (Table 2). Lemur species richness and relative abundance was highest in the non-degraded S01 site (n = 9 and 1.52 ± SE 0.11, respectively) and diminished as degradation increased (Table 2).

### Carnivore consumption/hunting

The highest total human consumption of carnivores occurred at less degraded sites (S02 and S03) whereas the lowest rates occurred at the highly degraded site (S06; Table 3). Across the four study sites the data indicate that all hunting rates, particularly trapping, purchasing, and hunting with dogs, are higher for less degraded sites (S01 and S02) compared to more degraded sites (S03 and S06). The exotic small Indian civet was the most heavily consumed carnivore across the four sites (n = 90 individuals/year) followed by native ring-tailed vontsira (n = 58 individuals/year) and fosa (n = 31 individuals/year). Spotted fanaloka was the least consumed carnivore across the four surveyed sites (n = 5 individuals/year); however, insufficient data on broad-striped vontsira at two sites prevented comparison with co-occurring carnivores. Across the four sites, small Indian civet and ring-tailed vontsira were also consistently the highest trapped species per year (23 and 12, respectively), the highest number purchased per year (3 and 1, respectively), and highest number hunted with dogs per year (9 and 7, respectively).

**Table 3:** Hunting results, including the total number of animals consumed, trapped, purchased, and hunted with dogs per village per year, for five native and two exotic (bold) carnivores. A subset of households across four villages (S01, S02, S03, and S06) were surveyed an average of three times between 2005 and 2011. Number of households per village ranged from 12 to 177.

Total per village per year	Site	Fosa (*C*. *ferox*)	Spotted fanaloka (*F*. *fossana*)	Falanouc (*E*. *goudotii*)	Ring-tailed vontsira (*G*. *elegans*)	Broad-striped vontsira (*G*. *fasciata*)	Feral cat (*Felis sp*.)	Small Indian civet (*V*. *indica*)	Total
# Consumed ^a^	S01	3	<1	1	8	1	1	10	24
	S02	16	2	5	25	-	5	47	100
	S03	7	2	3	18	-	1	23	54
	S06	5	1	1	7	1	1	10	26
# Trapped ^b^	S01	<1	0	1	4	1	<1	2	8
	S02	4	<1	2	4	-	0	14	24
	S03	0	0	0	3	-	0	3	6
	S06	3	0	1	1	0	0	4	9
# Purchased ^c^	S01	0	0	0	<1	0	0	1	1
	S02	<1	1	1	0	-	0	2	4
	S03	0	0	0	0	-	0	0	0
	S06	0	0	0	0	0	0	0	0
# Hunted with Dogs ^d^	S01	0	0	0	2	0	0	1	3
	S02	0	1	0	4	-	1	5	11
	S03	0	0	0	<1	-	0	<1	0
	S06	0	0	0	1	<1	0	3	4

^a^—Total number of individuals consumed per year where individuals were acquired via trapping, purchasing, hunting, or other additional measures.

^b^—Total number of individuals actively trapped per year; contributed to number consumed, but not correlated.

^c^—Total number of individuals purchased per year from local market or from an individual within their village; contributed to number consumed, but not correlated.

^d^—Total number of individuals actively hunted with personal domestic dog per year; contributed to number consumed, but not correlated.

### Occupancy: Landscape

Spotted fanaloka had the highest occupancy (0.70 ± SE 0.07) of any native carnivore across the landscape, followed closely by fosa (0.68 ± SE 0.08), while the domestic dog had the highest occupancy (0.61 ± SE 0.07) for any exotic carnivore (Table 4). Brown-tailed vontsira had the lowest occupancy (0.25 ± SE 0.09) of any native carnivore across the landscape while small Indian civet had the lowest (0.11 ± SE 0.03) for any exotic carnivore (Table 4). Domestic dog had a higher landscape occupancy estimate than four of the six native carnivore species while feral cat had a higher landscape occupancy estimate (0.37 ± SE 0.08) than three native species across the landscape (Table 4).

**Table 4:** Top ranking (ΔAIC < 2.0) landscape single-season occupancy models and the estimate of occupancy (Ψ) and detection (p) for six native and three exotic (bold) carnivore species across the Masoala-Makira landscape, NE Madagascar. Photographic surveys were conducted from 2008–2012 and were combined across all seven sites. Relationships (direction and magnitude) denoted by the betas for occupancy and detection are provided in Appendix I and II (respectively).

Scientific Name	Model *	AIC	Delta AIC	AIC wt.	Model Likelihood	K**	Ψ (SE)	p (SE)
Fosa	Ψ(TrType+PhysDes), p(%Matrix+Cover)	856.26	0	0.74	1.00	6	0.68 (0.08)	0.15 (0.02)
Spotted fanaloka	Ψ(VI+Cat), p(TotEdge+#Patches)	748.69	0	0.46	1.00	6	0.70 (0.07)	0.17 (0.02)
	Ψ(CanHt+Cat), p(TotEdge+#Patches)	748.83	0.14	0.43	0.93	6		
Falanouc	Ψ(Bird+VI), p(%Matrix+Village)	466.64	0	0.64	1.00	6	0.31 (0.07)	0.20 (0.05)
Ring-tailed vontsira	Ψ(Bird+Under), p(TotEdge+Cat)	468.44	0	0.71	1.00	6	0.48 (0.08)	0.10 (0.03)
Broad-striped vont.	Ψ(SmMamm+Village), p(Human+Camera)	415.25	0	0.61	1.00	6	0.28 †	0.06 (0.01)
Brown-tailed vont.	Ψ(Bird), p(Human)	125.25	0	0.99	1.00	4	0.25 (0.09)	0.05 (0.02)
**Domestic dog**	Ψ(Human+SmMamm), p(Human+TrType)	851.02	0	0.98	1.00	6	0.61 (0.07)	0.27 (0.02)
**Feral cat**	Ψ(Bird+Cover), p(TotEdge)	337.27	0	0.53	1.00	5	0.37 (0.08)	0.08 (0.02)
	Ψ(Village+Cover), p(TotEdge)	337.54	0.27	0.46	0.87	5		
Small **Indian civet**	Ψ(Village), p(%Rain)	237.91	0	0.35	1.00	4	0.11 (0.03)	0.05 (0.02)
	Ψ(Village), p(%Rain+Village)	239.62	1.71	0.15	0.43	5		

* = variable descriptions for each model provided below

^†^ = naïve estimate of occupancy due to the model not fitting the data (GOF > 0.05; c-hat > 3.0)

TrType = trail type (ordered widest to smallest); PhysDes = physical description (ordered ridge, valley, slope); %Matrix = percent of landscape consisting of non-forest, cultivated area; Cover = percent canopy cover; VI = small Indian civet *(Viverricula indica)* trap success; Cat = feral cat trap success; TotEdge = total edge (in meters per hectare); #Patches = total number of rainforest, degraded forest, and matrix patches within the camera grid buffer; CanHt = average canopy height; Bird = bird trap success (all species); Village = average distance of each camera station to the nearest village; Lemur = lemur relative abundance (all species); Under = total understory cover from 0 to 2.0 m; SmMamm = small mammal trap success (all species); Human = human trap success; Camera = camera model combination (Reconyx, Moultrie, Cuddeback, DeerCam brands); %Rain = percent of landscape consisting of rainforest cover.

** K = number of parameters within the model.

**Fig 2:**
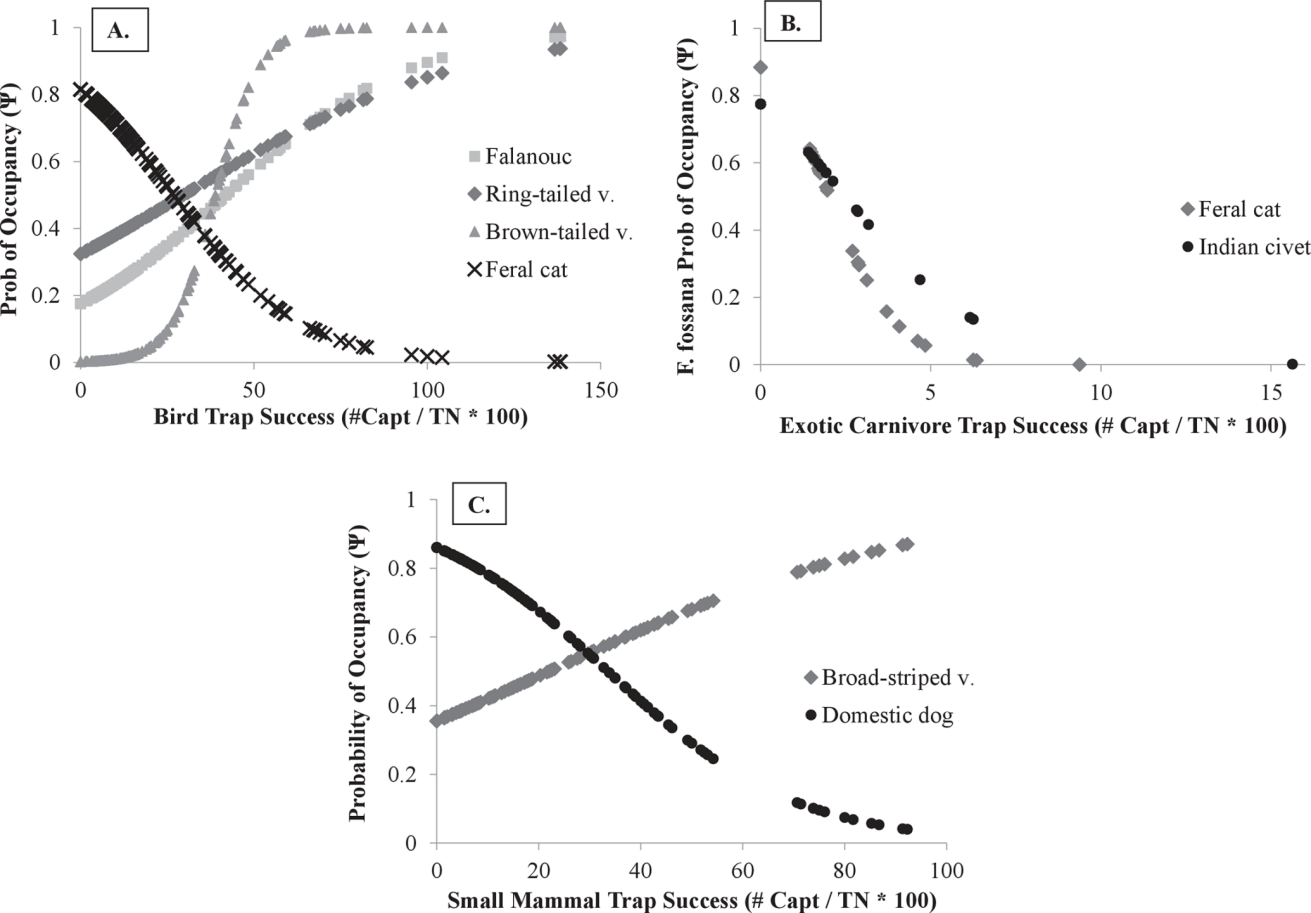
Single-season occupancy estimates for native and exotic carnivores. Probability of occupancy for: A) multiple native carnivores (grey symbols) and the exotic feral cat (black symbols) as a function of bird trap success (number of captures/trap night * 100); B) broad-striped vontsira (grey symbol) and exotic domestic dog (black symbol) as a function of small mammal trap success; and C) spotted fanaloka (*Fossa fossana*) as a function of feral cat (gray) and small Indian civet (black) trap success based on regression coefficients (β) resulting from landscape level single-season occupancy models across all seven sites combined.

We found bird trap success to be the most important variable for predicting carnivore occupancy across species (Table 4; S1 Table) with a positive relationship for three natives and a negative relationship for feral cat (Fig 2a). Further, we found a similar relationship between small mammals and native broad-striped vontsira (positive) and domestic dogs (negative) (Fig 2b; S1 Table). Additionally, the presence of feral cats and small Indian civets negatively affected spotted fanaloka occupancy (Fig 2c). Human trap success was the most common variable for predicting carnivore detection, negatively influencing the detection of two native species (broad-striped vontsira and brown-tailed vontsira) and positively influencing domestic dog detection. Percent matrix (percentage of the landscape consisting of non-forest and/or cultivated area) negatively affected fosa detection and positively affected falanouc detection. Total amount of edge negatively affected the detection of both natives (spotted fanaloka and ring-tailed vontsira) and exotics (feral cat) while distance to village (distance of each camera to the nearest village) negatively affected falanouc and positively affected small Indian civet (Table 4; S2 Table).

### Occupancy: Site Specific

**Fig 3:**
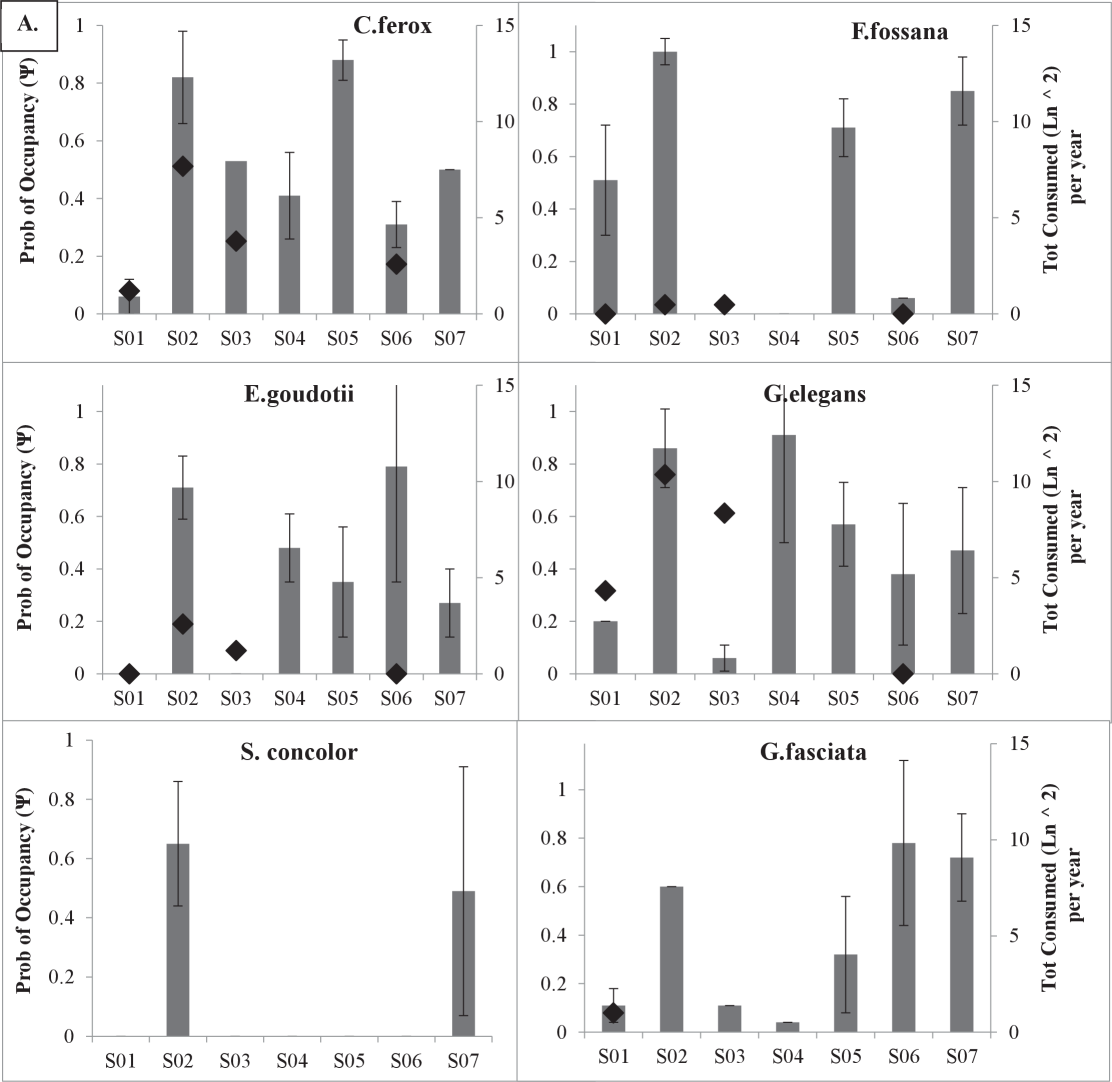
Site-specific single-season occupancy for native and exotic carnivores across the landscape. Site-specific occupancy estimates (± SE) for each native A) and exotic B) carnivore species across the seven study sites, ranked from least degraded (S01) to most degraded (S07), with the estimated total number of animals consumed per year (diamond; natural log squared) by site on secondary axis. The * indicates the naïve occupancy estimate was used.

When exploring within species trends in single-season occupancy across the continuum of degradation, we found no clear patterns in native carnivore occupancy rates moving from non-degraded to degraded forest (Fig 3a). However, all three exotic carnivore species show an increase in site specific occupancy with increases in degradation (Fig 3b).

### Occupancy: Hunting

We found a positive relationship between metrics of carnivore consumption and hunting and carnivore occupancy at all four sites (for example, see fosa in Fig 3a; Table 5). Thus, high carnivore occupancy is associated with high rates of carnivore consumption and all three metrics of hunting (Table 5). In addition, we found a positive relationship between hunting rates and carnivore detection for all species except ring-tailed vontsira and feral cat (Table 5).

**Table 5:** Top ranking (ΔAIC < 2.0) single-season occupancy models and estimates of occupancy (Ψ) and detection (p) across the four sites with hunting data (excluding any additional variables) for four native and one exotic (bold) carnivore species, across the Masoala-Makira landscape, NE Madagascar. Photographic surveys were conducted from 2008–2012 and bushmeat surveys from 2005–2011. Covariates in bold signify a positive relationship and non-bold a negative relationship with occupancy and/or detection. Betas (SE) for occupancy and detection (p) also included.

Common Name	Model	AIC	Delta AIC	AIC wt.	# Par.	Beta (Ψ)	Beta (p)	Ψ (SE)	p (SE)
Fosa	Ψ(.), p(**#Purchased**)	317.11	0	0.59	3	.	0.74 (0.16)	0.74 (0.12)	0.10 (0.02)
	Ψ(**#Consumed**), p(**#Purchased**)	318.05	0.94	0.37	4	0.52 (0.42)	0.64 (0.18)		
Spotted fanaloka	Ψ(**#Trapped**), p(**#Trapped**) ^a^	309.59	0	0.97	4	12.65 (23.36)	1.11 (0.23)	0.40 (0.06)	0.26 (0.05)
Falanouc	Ψ(.), p(**#Purchased**) ^b^	228.95	0	0.45	3	.	1.06 (0.20)	0.69 (0.13)	0.09 (0.02)
	Ψ(**#Purchased**), p(**#Purchased**)	229.04	0.09	0.43	4	0.91 (0.46)	0.60 (0.33)		
Ring-tailed vontsira	Ψ(**AllDogHunts**) ^c^, p(#Purchased)	186.08	0	0.95	4	4.02 (1.45)	-0.81 (0.27)	0.48 (0.08)	0.12 (0.04)
**Feral cat**	Ψ(.), p(#Consumed)	216.07	0	0.96	3	.	-2.31 (0.62)	0.78 (0.15)	0.07 (0.02)

^a^—#Trapped was correlated with #Purchased for this model set, thus #Purchased was not used.

^b^—#Purchased was correlated with #DogHunts for this model set, thus #DogHunts was not used.

^c^—AllDogHunts = total number of wildlife species hunted with domestic dogs per year

## Discussion

Our findings reveal increases in exotic carnivores as degradation increases and high hunting and consumption rates across the landscape, which are likely negatively influencing this endemic ecosystem and the biodiversity inhabiting it. However, we found mixed results regarding native carnivores and increasing degradation. In particular, our results show that increases in anthropogenic disturbance (ex. human trap success, distance to village, and distance to forest edge) are positively related to exotic carnivore occupancy and encounter rates, as well as increasing fragmentation (more edge, more patches, less core rainforest), which has been shown in other studies to reduce gene flow, impede movement across the landscape, reduce population size, and increase the likelihood of disease and pathogen transfer for wide-ranging carnivores [8,12,63,64]. In addition, we found negative relationships among exotic carnivores and native prey species, and negative trends in lemur species richness and relative abundance as degradation increases. We suggest that increases in exotic carnivores will result in further decreases in population density and species richness of native lemur, bird, and small mammal groups across Madagascar’s eastern rainforest region; however, additional surveys which account for temporal trends and confounding variables are needed to effectively test this hypothesis.

Our study consisted of single-season surveys over a gradient of degraded sites in which we found higher native carnivore occupancy at moderately degraded sites, corroborating other studies that have shown moderate habitat disturbance to have positive effects on some small-bodied carnivore populations [10,65,66]. However, native carnivores still face pressure across these degraded sites as evidenced by: 1) the absence of numerous carnivores at one or more heavily degraded forest sites; 2) a strong influx of exotic species with increasing degradation; and 3) intense hunting pressure, particularly where carnivore occupancy is highest. We suggest these findings for native carnivores may indicate the occurrence of extinction debt (i.e. impending extinction due to on-going and/or past events) across the landscape [67]. This hypothesis is further supported by strong decreases in native carnivore occupancy and strong increases in exotic carnivore occupancy at multiple long-term study sites over a six-year period (Farris, unpublished data). Additional longitudinal studies (across sites and years) are needed to further test these predictions.

The low estimates of native carnivore encounter rates and occupancy at the least degraded site (S01) may be attributed to the placement of cameras along newly cut trails, given the absence of existing trails at this contiguous forest site. The importance of sampling along existing trails is demonstrated both in the existing literature [45,55] and in the fosa models that show a decrease in occupancy (negative regression coefficient) as trails decrease in width (Table 4, Appendix I). However, this site also lies at a much higher elevation compared to the other sites and this is likely to have an effect on the habitat characteristics and co-occurring wildlife populations at this site. We need additional surveys at other high elevation sites across this region to better understand the effects elevation may have on carnivore populations in Madagascar.

Spotted fanaloka and fosa had the highest estimates of occupancy across the landscape; however, spotted fanaloka exhibits one of the most restricted ranges being absent at two sites and exhibiting very low occupancy at another, while fosa was found at all study sites and exhibited higher occupancy where trails are widest and well maintained. Brown-tailed vontsira had the most restrictive range and lowest occupancy of any native carnivore; these findings shed further light on the overall rarity of this native carnivore which appears to be a low elevation specialist (IUCN 2015) and we suggest it may face the greatest threat from on-going forest loss and fragmentation, as lower elevation forest is being lost at a faster rate [30,68].

Our findings for exotic carnivores, namely their positive association with degradation and that dogs and cats have higher occupancy than half the native carnivores, are congruent with those by Gerber et al. [13,14] which highlighted increases in exotic carnivores in fragmented and degraded forest sites and negative relationships in occupancy and temporal interactions between native and exotic carnivores. The negative relationship between exotic carnivores and native species (including birds, small mammals, spotted fanaloka, and lemurs) is particularly alarming due to the importance of native birds and small mammals for the diet of multiple native carnivores [36], the important ecosystem services these native species may provide, and the ability of exotics to cause the local extirpation of bird and/or small mammal populations within various habitats worldwide [69–71]. Lemur encounter rates decreased where exotic carnivore occupancy was high, corroborating previous findings by Farris et al. [39] of negative interactions between multiple lemurs and exotic carnivores. With approximately 94% of Madagascar’s lemur species classified as threatened [72], the strong increase in exotic carnivore presence in multiple habitats across Madagascar, and the corresponding apparent negative effects on lemur populations, these trends demand immediate action.

In addition to the effects of habitat degradation and exotic species presence, our results provide further insight into the widespread, unsustainable hunting trends and exceedingly high rates of consumption (> 10 individuals per year) threatening the carnivore community across the Masoala-Makira landscape [23,25]. The exotic small Indian civet and native ring-tailed vontsira were the two most consumed, trapped, hunted, and purchased carnivores, likely reflecting their increased activity and/or density in matrix and edge habitat near anthropogenic areas [36,73,74] where hunting pressure is more intense (Golden, unpublished data). This hypothesis is further supported by our findings which reveal higher hunting rates for wide-ranging species that use matrix habitat (fosa and feral cat) compared to the more restrictive species (spotted fanaloka and falanouc) which have been shown in previous studies to show reliance on contiguous, non-degraded forest [13,36,44,75,76].

Given that high hunting and consumption rates of wildlife across this region are likely leading to population declines for multiple carnivore, lemur, and small mammal species [23,25], we expected to find lower native carnivore occupancy and detection at sites where hunting rates were highest; however, the opposite was observed at the four study sites we surveyed. We suggest this reflects the past “hunting out” of more degraded sites, with local hunters targeting non-degraded forest sites where wildlife populations occur at higher densities in their effort to increase the number of successful hunts. In this case, where hunting is nearly entirely passive (i.e. traps and snares), animals are harvested in a density-dependent fashion in accordance with their prevalence in the forest. While this form of passive and/or opportunistic hunting has been observed in other areas of Madagascar [43], additional work in other regions of Madagascar has shown hunting to be commercially motivated [77]. Our findings highlight the need for additional studies to compare the effects of active versus passive hunting efforts on carnivore populations worldwide. An understanding of how hunting influences carnivore population dynamics across the tropics remains un-studied and is needed to properly estimate sustainability for management and conservation purposes [21,22]

The findings of this study and other similar works demand attention from conservation managers working to protect threatened species worldwide. To combat fragmentation and its negative effects on wildlife, we suggest managers focus efforts on developing community-led management strategies to conserve large tracts of contiguous forests. Further, implementing reforestation projects to reconnect fragmented forests would diminish the effects of fragmentation to carnivore and co-occurring wildlife populations. Effective, well-regulated capture-removal programs for feral cats and domestic dogs must be considered in Madagascar and in similar protected areas worldwide to diminish the threat exotic species pose to native wildlife. While such programs have been met with mixed results [70,78–81] and may be expensive, the control of exotic carnivores is important for the successful protection and management of native wildlife. To further combat the impact of exotic carnivores on native wildlife, we suggest managers develop educational materials to inform people living near these protected areas about the dangers their domestic pets pose to local wildlife, encourage people to leave pets at home when traveling to nearby forests, and provide spay/neuter and vaccination services to reduce the local dog and cat populations. Finally, similar to other areas in the world, bushmeat consumption in Madagascar, while unsustainable, is tied to human health and the local economy [25]. Hunting and consumption of exotic carnivores may act to control the spread of these exotic species; however, these efforts are likely to only apply to the small Indian civet and/or feral cat as local taboos or *fady* might limit or prevent the consumption of domestic/feral dogs (Kim Reuter, personal communication). Given the widespread preference for domestic meat over bushmeat [25,43,82] we recommend managers in Madagascar and in protected areas worldwide consider domestic fowl breeding programs to replace wildlife bushmeat hunting and consumption, as is currently being conducted by the Wildlife Conservation Society, Madagascar Program across the Makira Natural Park.

## Conclusion

Our results provide additional insight into the threat posed to carnivore communities worldwide from widespread anthropogenic pressures, namely fragmentation, exotic carnivores, and hunting. Our findings contribute to the growing body of knowledge on the negative effects of exotic carnivores on native wildlife found worldwide [17,69–71,78,83–85] and add to the discussion on the drivers of exotic carnivore invasions and resulting conservation issues [86–89]. In particular, the trends in exotic carnivore occupancy and their negative relationship with native carnivores, lemurs, small mammals, and birds demonstrated by this study and others throughout Madagascar [13,14,18,19,39] merit immediate, targeted conservation and management plans to reduce the influx of exotic carnivore species in Madagascar’s eastern rainforests. We recommend an effective capture-removal program for feral cats and domestic dogs to diminish the threat posed to native wildlife, both in Madagascar and worldwide. Hunting presents an intense, augmenting pressure for carnivores across this region and in multiple other tropical countries. We recommend a holistic wildlife conservation and local livelihoods approach to address the threat of hunting and ensure long-term success in conserving native wildlife. If unchecked these anthropogenic pressures are likely to result in the local extirpation of numerous native carnivore, lemur, bird, and small mammal species across the Masoala-Makira landscape. Finally, these various anthropogenic pressures are known to negatively affect carnivore populations worldwide, thus these findings, including how these variables act synergistically across the landscape, have wide-ranging implications for managers working to conserve carnivore populations worldwide.

## Supporting Information

S1 Table Regression coefficients for top single-season occupancy models. Logistic regression coefficients, β (SE) for top occupancy models for each native and exotic (bold) carnivore species across the Masoala-Makira landscape, Madagascar. Sampling occurred from Aug 2008 –October 2012. Bold font signifies support for relationship between the variable and species occupancy (i.e. CI does not overlap zero).
(PDF)

S2 Table Regression coefficients for top detection models. Logistic regression coefficients, β (SE) for detection probabilities resulting from top landscape single-season occupancy models for each native and exotic (bold) carnivore species across the Masoala-Makira landscape, Madagascar. Sampling occurred from Aug 2008 –October 2012. Bold font signifies support for relationship between the variable and species detection (i.e. CIs do not overlap zero).
(PDF)
