# Supplementary material for: Hunting, Exotic Carnivores, and Habitat Loss: Anthropogenic Effects on a Native Carnivore Community, Madagascar
Source: PLoS One. 2015 Sep 16;10(9):e0136456. doi: 10.1371/journal.pone.0136456 (PMC4573327; doi:10.1371/journal.pone.0136456)
Supplement: S1 Table — Logistic regression coefficients, β (SE) for top occupancy models for each native and exotic (bold) carnivore species across the Masoala-Makira landscape, Madagascar. Sampling occurred from Aug 2008 –October 2012. Bold font signifies support for relationship between the variable and species occupancy (i.e. CI does not overlap zero). (PDF) [file pone.0136456.s001.pdf]

Appendix I.

| Parameter | Fossa (C. ferox) | Malagasy civet (F. fossana) | Falanouc (E. goudotii) | Ring-tail vontsira (G. elegans) | Broad-stripe vontsira (G. fasciata) | Brown-tail vontsira (*S. concolor*) | Domestic dog (*C. familiaris*) | Wild/feral cat (*Felis sp.*) | Indian civet (*V. indica*) |
|---|---|---|---|---|---|---|---|---|---|
| Intercept | **2.79 (0.75)** | **1.04 (0.48)** | **-0.66 (0.28)** | **-0.80 (0.34)** | **-0.83 (0.39)** | **-1.93 (0.91)** | 0.34 (0.56) | **-2.01 (0.70)** | **-1.75 (0.63)** |
| Cat | - | **-2.65 (1.0)** | - | - | - | - | | - | - |
| Indian civet | | **-1.20 (0.52)** | 0.72 (0.37) | - | - | - | - | - | - |
| TrType | **-2.29 (0.60)** | - | - | - | - | - | - | - | - |
| Bird | - | | **1.00 (0.41)** | 0.78 (0.43) | - | 4.54 (3.26) | - | **-4.05 (0.99)** | - |
| DistVillage | - | - | - | - | **-0.93 (0.38)** | - | - | - | **-1.59 (0.87)** |
| Human | - | - | - | - | - | - | 3.55 (2.09) | - | - |
| SmMamm | - | - | - | - | 0.81 (0.46) | - | **-0.92 (0.34)** | - | - |
| CanCover | - | - | - | - | - | - | - | <0.01 | - |
| PhysDes | 0.09 (0.32) | - | - | - | - | - | - | - | - |
| Understory | - | - | - | <0.01 | - | - | - | - | - |
