# Supplementary material for: Hunting, Exotic Carnivores, and Habitat Loss: Anthropogenic Effects on a Native Carnivore Community, Madagascar
Source: PLoS One. 2015 Sep 16;10(9):e0136456. doi: 10.1371/journal.pone.0136456 (PMC4573327; doi:10.1371/journal.pone.0136456)
Supplement: S2 Table — Logistic regression coefficients, β (SE) for detection probabilities resulting from top landscape single-season occupancy models for each native and exotic (bold) carnivore species across the Masoala-Makira landscape, Madagascar. Sampling occurred from Aug 2008 –October 2012. Bold font signifies support for relationship between the variable and species detection (i.e. CIs do not overlap zero). (PDF) [file pone.0136456.s002.pdf]

Appendix II.

Parameter	Fossa (C. ferox)	Malagasy civet (F. fossana)	Falanouc (E. goudotii)	Ring-tail vontsira (G. elegans)	Broad-stripe vontsira (G. fasciata)	Brown-tail vontsira (*S. concolor*)	Domestic dog (*C. familiaris*)	Wild/feral cat (*Felis sp.*)	Indian civet (*V. indica*)
Intercept	**-0.92 (0.16)**	**-3.10 (0.48)**	**-2.44 (0.33)**	**-0.90 (0.32)**	**-3.84 (0.66)**	-10.92 (6.68)	**1.48 (0.67)**	**-1.58 (0.22)**	-0.28 (0.71)
CanCover	<0.01	-	-	-	-	-	-	-	-
%Matrix	-0.10 (0.18)	-	**0.08 (0.02)**	-	-	-	-	-	-
%Rain	-	-	-	-	-	-	-	-	**-0.03 (0.01)**
TotEdge	-	**-6.30 (0.81)**	-	**-5.42 (1.21)**	-	-	-	**-4.10 (1.26)**	-
Human	-	-	-	-	**-0.75 (0.37)**	-19.08 (15.06)	**1.16 (0.12)**	-	-
TrailType	-	-	-	-	-	-	**-2.19 (0.65)**	-	-
CameraType	-	-	-	-	**7.14 (2.71)**	-	-	-	-
Cat	-	-	-	**-0.98 (0.46)**	-	-	-	-	-
DistVillage	-	-	-0.47 (0.39)	-	-	-	-	-	-
#Patches	-	**4.49 (0.57)**	-	-	-	-	-	-	-
